# Influence of sanitation facilities on diarrhea prevalence among children aged below 5 years in flood-prone areas of Bangladesh: a multilevel analysis

**DOI:** 10.1007/s11356-023-29373-0

**Published:** 2023-08-21

**Authors:** Michiko Kikuchi

**Affiliations:** Graduate School of Frontier Sciences, The University of Tokyo, 5-1-5 Kashiwanoha, Kashiwa City, Chiba Prefecture 277-8561 Japan

**Keywords:** Diarrhea, Child morbidity, Sanitation, Flood risk, Demographic and health survey, Multilevel linear probability model

## Abstract

Although the improvement of sanitation facilities has been a major contributor to improving public health, it is not guaranteed to prevent negative health outcomes. This is especially true in areas affected by severe natural disasters, such as flooding or extreme rainfall. Previous studies have examined the association between catastrophic natural disasters and negative health outcomes. However, studies on disaster-prone areas are limited. This study focused on the impact of flood risks and examined whether the improvement of sanitation facilities would be sufficient to suppress the prevalence of diarrhea in flood-prone areas. Two secondary datasets including geodata on flood-prone areas were used for the analysis: one each was obtained from the Bangladesh Demographic and Health Survey and Bangladesh Agricultural Research Council. Two models with categorizations of sanitation facilities based on containment type and excreta flow were applied for analysis. Results showed that the severe flood-prone areas and “diffused” type of sanitation, where the feces are diffused without any containment, had significant positive associations with diarrhea prevalence; however, the interaction between them was negative. Moderate flood-prone areas had a significant positive association with diarrhea prevalence; however, the interaction with unimproved sanitation, which includes containment without clear partition from feces, was significantly negative. These findings indicate that improved sanitation or containment type of sanitation may not positively contribute to the prevention of diarrhea in these severe- and moderate-flood prone areas. The urgent need for alternative sanitation technologies should be addressed in flood-prone regions.

**Supplementary Information:**

The online version contains supplementary material available at 10.1007/s11356-023-29373-0.

## Introduction

Although improving sanitation facilities has been a major contributor to the improvement of public health in resource-limited regions, such as low- and middle-income countries (Cha et al. 2017; Chowdhury et al. 2015; Contreras et al. 2022; Dagnew et al. 2019; Local Burden of Disease WaSH Collaborators, 2020; Luby et al. 2018; Null et al. 2018; Omotayo et al. 2021; UNICEF and WHO 2019), there has been some debate surrounding the negative effects of improved sanitation facilities, such as pit latrines, on the environment. Dzwairo et al. (2006) detected high conductivity in the ground water at a lateral distance of approximately 25 m from a pit latrine in rural areas of Zimbabwe, which is an indicator of the impact of the pit latrine. Islam et al. (2016) investigated groundwater contamination with a focus on different hydrogeological conditions of floodplains in Bangladesh and showed that where the aquitard was thin, the contamination in the groundwater was detected in a broader area, both horizontally and vertically, from the location of pit latrines. Dey et al. (2017) analyzed four hydrological regions of Bangladesh and showed that the groundwater contamination was higher in the northwestern regions compared to the other regions during the wet season based on the detection of *Escherichia coli*, which indicates human excreta. Thus, although pit latrines are a safe option, geological, seasonal, environmental, and climatological factors may influence the effect of sanitation facilities on health and are therefore important factors to consider.

Floods are a commonly occurring natural disaster that affect human health. Previous studies have argued that flood events (Chowdhury et al. 2018; Hashimoto et al. 2014; Hashizume et al. 2008a, b; Zhang et al. 2019) and recurrent disasters (Joshi et al. 2011) affect the prevalence of diarrhea. The risk of water- and vector-borne diseases is high in flood-affected areas (Abbas and Routray 2014; Levy et al. 2016), and diarrhea prevalence increases during periods of flooding (Schwartz et al. 2006).

Floods also affect sanitation facilities, causing the spread of waterborne infectious diseases. A study conducted in the flood-prone regions of Nicaragua revealed that overflowing sanitation facilities increased the likelihood of diarrhea prevalence (Denslow et al. 2010). Studies on the geological features of flood-prone areas have shown that these areas generally occupy large alluvial plains in lowlands (Wesselink et al. 2015), which implies that the infrastructure in these areas is more susceptible to disruption or deterioration when compared with the infrastructure in non-flood-prone areas (Mukherjee et al. 2009). Considering the state of flood-prone environments, appropriate sanitation facilities should be developed, and existing sanitation technology should be updated and implemented. A review by Borges Pedro et al. (2020) discussed the appropriate sanitation technologies in flood-prone areas worldwide; however, the health impacts in those areas have not been fully explored.

Bangladesh, which occupies the world’s largest river delta, is one of the most flood-prone countries globally (Mukherjee et al. 2009; Wesselink et al. 2015). It experiences various flood types annually due to the different geological and climatological features of the region (Dewan 2015). Three main flood types, namely river floods, flash floods, and tidal surges, are observed in Bangladesh (Bangladesh Agricultural Research Council: BARC 2022; Table S1). River floods are caused by the overflow of riverbanks or extreme rainfall and are mostly observed during the monsoon season (June to October). Flash floods are mainly observed in north-eastern and south-eastern hilly areas, where abnormal amounts of water surge from hillsides between early April and May. Tidal surges are observed in coastal areas and are caused by cyclones between June and September. Floods in Bangladesh are observed every year, but catastrophic severe flooding events are only observed every few years (BWDB 2017).

The geological features of Bangladesh also affect the infrastructure in the area. Bangladesh has three major rivers that shape the Bengal Delta; these are Ganga, Brahmaputra, and Meghna, which merge into the Bay of Bengal. These rivers have deposited floodplains and shaped the Barind and Madhupur Pleistocene tracts in the central and northwestern areas, respectively (Shamsudduha and Uddin 2007). Floodplains or alluvium absorb water from floods, especially in lower land basins during the monsoon season. This enriches the soil for agricultural purposes but may cause infrastructure to become unstable and deteriorate, especially in severe flood-prone areas (Brammer 1990; Umitsu 1993). Therefore, it is crucial to evaluate the appropriateness of infrastructure construction in these areas. However, there are few studies on infrastructure, especially sanitation facilities, in flood-prone areas of Bangladesh.

In 2017, Bangladesh was declared “open defecation free” (UNICEF and WHO 2019); however, most urban and rural areas, except for some areas of mega cities such as Dhaka (Yin et al. 2021), do not yet have sufficient sewer systems or treatment plants to serve the entire population, which means that most people still rely on on-site sanitation systems such as pit latrines. Although pit latrines are an affordable option for low-income households, particularly in rural areas, the negative effects of pit latrines on floodplains have not yet been fully examined.

Bangladesh has experienced remarkable success in the health sector, albeit with high morbidity rates and low use of health services (Chowdhury et al. 2013). For example, the number of diarrhea-related deaths dropped significantly between the years 2007 and 2016 (Government of the People’s Republic of Bangladesh, Ministry of Health and Family Welfare: GOB-MHFW 2019). However, the diarrhea morbidity rate remains high. Therefore, focusing on diarrhea morbidity, this study aimed to examine the effect of sanitation facilities on the incidence of diarrhea in children below 5 years of age, living in disaster-prone areas of Bangladesh with various levels of flood risk, as well as the relationship between sanitation facility types and flood risk. Furthermore, the nationwide spatial distribution of diarrhea prevalence was evaluated to identify focus areas for reducing diarrhea-related morbidity in Bangladesh. Considering the effect of environmental factors on sanitation facilities can help to assess the suitability of these facilities for disaster-prone areas of Bangladesh.

## Methods

### Data

Two secondary datasets were aggregated in this study. The first dataset was the Bangladesh Demographic and Health Survey (BDHS) conducted in the years 2011 (BDHS 2011), 2014 (BDHS 2014), and 2017–2018 (BDHS 2017–2018) (The DHS Program). This survey was implemented by the National Institute of Population Research and Training and Mitra and Associates of Dhaka, to obtain information, including details on caregivers, geodata, and diarrhea incidence in children < 5 years old, which is the target population of this study. Survey data were based on a stratified two-stage cluster design, wherein each sample was represented at both the national and semi-national levels. Each respondent represented a data point within a cluster, which in turn represents the buffer in the dataset. Each cluster consisted of data from respondents who live within its buffer zone. Cluster data also included average population data calculated within the 2 km (urban area) or 10 km (rural area) buffer surrounding the cluster location (Burgert et al. 2013). These data were combined using geodata.

In this study, the dependent variable was diarrhea incidence in children < 5 years of age obtained from the results of the DHS survey. When questioned if the child experienced diarrhea in the 2 weeks prior to the survey, the parent or caregiver’s response would be either “Yes,” “No,” or “I don’t know.” This study only analyzed the “Yes” and “No” responses, as “I don’t know” responses may invite uncertainties due to the inaccurate recollections of respondents, who may be unaware of the child having diarrhea in the previous 2 weeks.

Independent variables were selected based on previous studies (Kamal et al. 2015; Sinmegn Mihrete et al. 2014). For individuals, the characteristics of each child, namely age, sex, mother’s age, mother’s education, access to sanitation facilities, access to drinking water facilities, wealth index, religion, and residence, were included in this study. The effect of sanitation facility type on diarrhea incidence was also examined. This study applied two types of categorizations of sanitation facilities for analysis: (1) Based on the definitions by the service ladders for sanitation provided by the Joint Monitoring Program (JMP) shown in Table S2 (UNICEF and WHO 2019), namely “improved,” “unimproved,” and “no facilities.” (2) Based on the post-defecation behavior of feces, namely “concentrated,” “diffused,” and “transferred” (Table S3). “Concentrated” refers to excreta held in an underground pit or tank for a specified period, which carries a probability of exposure of humans to pathogens. “Diffused” refers to excreta placed on the ground with a wider surface area rather than in a designated pit or tank. “Transferred” refers to excreta placed directly into sewer systems and transferred to wastewater treatment facilities. While the first categorization is based on how the facilities capture excreta without contacting humans, the second categorization focuses on the process after capturing excreta, that is, methods by which facilities contain or store excreta after capturing. The second categorization also considers the sanitation service chain, or fecal sludge management, which defines the comprehensive process of capturing human excreta and safely disposing it (Peal et al. 2015). Manga et al. (2022) show that the behavior of a pathogen after capture depends on the containment system, which may be affected by geographical and hydrological features. Therefore, this categorization was applied considering the geological and hydrological setting of Bangladesh (Dey et al. 2017). Based on the two categorizations, this study used two analytical models: Models I and II that used variables “containment type” and “excreta flow,” respectively (Table S4).

The second dataset included geodata of flood risk levels in Bangladesh, provided by BARC (2022). Depending on geological characteristics, BARC classifies floods as river floods, flash floods, and tidal surges. BARC also categorizes four levels of flood risk areas— “severe flood-prone (SFP),” “moderate flood-prone (MFP),” “low flood-prone (LFP),” and “not flood-prone (NFP)” (Tables S1 and S5, Fig. S1). This categorization is based on the frequency and severity of floods experienced in the last 10 years. The severity differs year on year; however, normal floods occur yearly and inundate approximately 25% of the total land area (Dewan 2015). No catastrophic floods were reported during the survey period (BWDB 2011, 2014, 2017, 2018). This study applied the four flood-prone levels to BDHS data using geodata.

Although this study targeted all regions in Bangladesh, some areas were excluded as they might have different health risk factors. According to the BDHS survey, the spatial distribution of diarrhea prevalence highlighted regional inequalities. Prevalence was high in the southern coastal area and parts of the eastern area of Bangladesh where people are exposed to the risk of severe tidal surge. On the other hand, the northwestern regions exhibited a relatively low diarrhea prevalence, with the exception of the western part of the Rajshahi division; however, this area is prone to a severe drought risk. Furthermore, in areas with a high population density, diarrhea might be caused by human-to-human contacts. Thus, the diarrhea cases in areas that have a high population density and are prone to severe drought were excluded from the study (Figs. S1 and S2). Densely populated areas with > 20,000 people were excluded using geodata of the primary sampling unit, which includes groups of approximately 10–20 households. Severe drought-prone areas were excluded by applying a drought risk area dataset provided by BARC (Fig. S2). Geodata on flood and drought risk are available in the Humanitarian Data Exchange (The Humanitarian Data Exchange 2021).

### Statistical analyses

The independent variables used in the analysis are shown in Table S6. Multivariate analysis was performed to examine all covariates and include community level as a random intercept. As BDHS involves two-stage stratified cluster sampling, the collected individual data at the community level are nested as a cluster. The structured multilevel random intercept model was implemented for such nested data. A multilevel logistic regression model is usually applied for binary dependent variables; however, this study used a linear probability model to assess the probability of sanitation and flood risk impact on diarrhea incidence. Linear regression is applied for non-binary dependent variables, but it can also be appropriate when the area of interest concerns the effectiveness of an estimation (Mood 2010). The interpretation is straightforward, and interaction terms are not influenced by random intercepts (Beier 2018; Mood 2010). However, the linear probability model may estimate values > 1 or < 0, which is not a fundamental problem if most coefficients fall within the range and have residual heteroscedasticity, because the dependent variable is binary, although this can be avoided by applying robust standard errors.

Confidence intervals (CIs) and coefficients (*β*) estimated in linear probability models were used for analysis in this study. As this study examined how sanitation type influences the effect of flood risk on diarrhea prevalence and vice versa, the interaction terms of sanitation type and flood risk were applied to the models.

For the spatial distribution of diarrhea prevalence, choropleth maps of each survey year were created in the aggregated BDHS. This study used districts as spatial enumeration units for diarrhea prevalence. The analyses were performed using the “lmerTest” package, and the map was created using the “sf” package for R version 4.1.2 (R Core Team 2021).

## Results

A total of 24,232 samples of children < 5 years of age were obtained from the BDHS for 2011, 2014, and 2017–2018. After removing all missing and excluded data, 14,404 data points were used for analysis, of which 697 were diarrhea cases. Table 1 shows the characteristics of BDHS target populations in 2011, 2014, and 2017–2018.

**Table 1:** Demographic data of target population based on BDHS reports of 2011, 2014, and 2017–18.

	Diarrhea = No	Diarrhea = Yes
Cases	13,707	697
BDHS survey year (%)		
2011	4829 (35.2)	245 (35.2)
2014	4274 (31.2)	208 (29.8)
2017-2018	4604 (33.6)	244 (35.0)
Child age (%)		
0–11 months	2348 (17.1)	148 (21.2)
12–23 months	2507 (18.3)	232 (33.3)
24–35 months	2690 (19.6)	128 (18.4)
36–47 months	2996 (21.9)	101 (14.5)
48–60 months	3166 (23.1)	88 (12.6)
Sex of child		
Male (%)	7070 (51.6)	392 (56.2)
Female (%)	6637 (48.4)	305 (43.8)
Sanitation: Containment type^a^ (%)		
Improved	7785 (56.8)	372 (53.4)
Unimproved	5922 (43.2)	325 (46.6)
Sanitation: Excreta flow^b^ (%)		
Concentrated	12,347 (90.1)	616 (88.4)
Diffused	1253 (9.1)	73 (10.5)
Transferred	107 (0.8)	8 (1.1)
Toilet shared/individual (%)		
Individual	7892 (57.6)	362 (51.9)
Shared	5212 (38.0)	300 (43.0)
No facilities	603 (4.4)	35 (5.0)
Water facilities (%)		
Improved	13337 (97.3)	683 (98.0)
Unimproved	370 (2.7)	14 (2.0)
Households with electricity		
No	5130 (37.4)	263 (37.7)
Yes (%)	8577 (62.6)	434 (62.3)
Households with a refrigerator (%)		
No	11357 (82.9)	589 (84.5)
Yes	2350 (17.1)	108 (15.5)
Literacy (%)		
Yes	8435 (61.5)	413 (59.3)
Some	1618 (11.8)	90 (12.9)
No	3654 (26.7)	194 (27.8)
Religion (%)		
Islam	12,482 (91.1)	651 (93.4)
Hindu	1144 (8.3)	44 (6.3)
Other religion	81 (0.6)	2 (0.3)
Mother’s education (%)		
High school	1198 (8.7)	55 (7.9)
Secondary school	5821 (42.5)	264 (37.9)
Primary school	4320 (31.5)	261 (37.4)
No education	2368 (17.3)	117 (16.8)
Mother’s age [mean (SD^*^)]	26.67 (5.88)	25.76 (5.54)
Residential area (%)		
Rural	9835 (71.8)	498 (71.4)
Urban	3872 (28.2)	199 (28.6)
Wealth index (%)		
Poorest	3804 (27.8)	200 (28.7)
Poorer	2909 (21.2)	163 (23.4)
Middle	2529 (18.5)	141 (20.2)
Richer	2465 (18.0)	94 (13.5)
Richest	2000 (14.6)	99 (14.2)
Division (%)		
Dhaka	2657 (19.4)	154 (22.1)
Chittagong	2433 (17.8)	121 (17.4)
Sylhet	2335 (17.0)	135 (19.4)
Khulna	1689 (12.3)	62 (8.9)
Barisal	1668 (12.2)	95 (13.6)
Rajshahi	1415 (10.3)	67 (9.6)
Rangpur	1510 (11.0)	63 (9.0)
Population of children < 5 years of age [mean (SD)]	1503.27 (3796.48)	1720.75 (4328.00)
Population density [mean (SD)]	1803.81 (2426.04)	1843.10 (2529.63)
Flood-prone area (%)		
NFP area	4756 (34.7)	246 (35.3)
LFP area	3140 (22.9)	116 (16.6)
MFP area	4151 (30.3)	223 (32.0)
SFP area	1660 (12.1)	112 (16.1)

^a^Sanitation type based on WHO/UNICEF Joint Monitoring Program sanitation ladder

^b^Sanitation type based on the post-defecation behavior of feces

^*^Standard deviation (SD)

The spatial distributions of diarrhea prevalence during each survey year are shown in Figs. S3–S5. Figure S6 indicates the average prevalence for all years. The districts of Manikganji in Dhaka, Nawabganji in Rajshahi, coastal areas of Barisal and Habiganji in Sylhet, and the western part of Chittagong reported high diarrhea prevalence.

The results of multilevel linear probability models are shown in Table S7; Table 2 shows only those results that were considered in this study. In Model I, containment type had no significant association with diarrhea incidence, with or without interactions between sanitation and flood risk categories, whereas the diffused sanitation type in Model II showed positive associations with diarrhea prevalence with interactions (*β* = 0.027, CI: 0.004, 0.053). Some flood-prone areas were associated with diarrhea incidence. In Model I without interactions, LFP areas showed significant negative associations with diarrhea incidence (*β* = −0.013, CI: −0.023, −0.003), and SFP areas exhibited significant positive associations with diarrhea incidence (*β* = 0.015, CI: 0.002, 0.028). Furthermore, in Model I with interactions, MFP areas showed significant positive associations with diarrhea incidence (*β* = 0.012, CI: 0.001, 0.024). In Model II, LFP areas showed significant negative associations with diarrhea incidence, both with (*β* = −0.113, CI: −0.02, −0.001) and without (*β* = −0.013, CI: −0.023, −0.003) interactions. SFP areas reported significant positive associations with the incidence of diarrhea both with (*β* = 0.022, CI: 0.008, 0.035) and without (*β* = 0.015, CI: 0.003, 0.027) interactions. Interaction effects were observed in both models. In Model I, unimproved sanitation and MFP interactions showed significant negative associations with diarrhea prevalence (*β* = −0.022, CI: −0.040, −0.005). In Model II, diffused sanitation type and SFP area interactions showed significant negative associations with diarrhea prevalence (*β* = −0.060, CI: −0.095, −0.023). As this study excluded high population density areas before analysis, transferred sanitation prevalence decreased. In addition to the main analysis, a sensitivity analysis was conducted for densely populated areas with > 20,000 people. The results of the sensitivity analysis were generally consistent with the results of the main analysis; however, some deviations were observed especially when the interaction term was included (Table S8).

**Table 2:** Multilevel linear probability models (Models I and II).

	Model I: Containment type				Model II: Excreta flow			
	without interaction		with interaction		without interaction		with interaction	
	*β*	95% CI	*β*	95% CI	*β*	95% CI	*β*	95% CI
Containment type (Model I)								
Improved	(reference)		(reference)					
Unimproved	0.003	(−0.004, 0.018)	0.015	(−0.0004, 0.026)				
Excreta flow (Model II)								
Concentrated					(reference)		(reference)	
Transferred					0.023	(−0.019, 0.068)	0.030	(−0.184, 0.079)
Diffused					0.004	(−0.012, 0.019)	0.027	(0.004, 0.053)
Flood type area								
Not flood-prone (NFP)	(reference)		(reference)		(reference)		(reference)	
Low (LFP)	−0.013	(−0.024, −0.003)	−0.009	(−0.022, 0.005)	−0.013	(−0.023, −0.003)	−0.113	(−0.02, −0.001)
Moderate (MFP)	0.003	(−0.005, 0.012)	0.012	(0.001, 0.024)	0.003	(−0.005, 0.012)	0.005	(−0.004, 0.014)
Severe (SFP)	0.015	(0.002, 0.028)	0.024	(−0.003, 0.030)	0.015	(0.002, 0.028)	0.022	(0.008, 0.035)
Interaction (Model I)								
Unimproved * LFP area			−0.009	(−0.029, 0.009)				
Unimproved * MFP area			−0.022	(−0.040, −0.004)				
Unimproved * SFP area			0.003	(−0.019, 0.028)				
Interaction (Model II)								
Transferred * LFP area							−0.025	(−0.121, 0.083)
Diffused * LFP area							−0.021	(−0.057, 0.012)
Transferred * MFP area							−0.066	(−0.224, 0.099)
Diffused * MFP area							−0.021	(−0.055, 0.012)
Transferred * SFP area							0.037	(−0.145, 0.210)
Diffused * SFP area							−0.060	(−0.095, −0.023)

Dependent variable: diarrhea = yes, Model I: multilevel linear probability model of Containment type, Model II: multilevel linear probability model of Excreta flow

## Discussion

This study examined the influence of various sanitation facility types and flood risk levels and their effect on diarrhea prevalence among children < 5 years of age in Bangladesh. Although previous studies have explored the effects of improved sanitation on health (Clasen et al. 2014; Knee et al. 2018; Patil et al. 2014; Sinha et al. 2017), this study added another categorization to sanitation facilities, focusing on transfer or storage of excreta in the environment. The study also analyzed how the interactions between flood risk and sanitation facilities affect diarrhea prevalence. Previous research has showed the influence of flood events or extreme weather on diarrhea prevalence in low- and middle-income countries (Carlton et al. 2014; Chowdhury et al. 2018; Dimitrova and Muttarak 2020; Hashimoto et al. 2014; Hashizume et al. 2008a, b; Milojevic et al. 2012). While these studies considered only specific events and regions, the current study has a wider scope as it analyzes flood frequency and severity using results of a national-level survey. This study showed how varying flood-prone levels influence diarrhea prevalence, depending on the type of sanitation facility available.

The multilevel linear probability model showed no association between the containment type in Model I and diarrhea prevalence with or without interactions; however, the interaction between unimproved facilities and MFP area exhibited significant positive associations with diarrhea prevalence. In Model II, diffused sanitation type showed significant positive associations with diarrhea prevalence with interactions, whereas the interaction between diffused sanitation type and SFP area showed significant negative associations with diarrhea prevalence. This result may indicate that diffused sanitation type reduces the possibility of contact with pathogens in SFP areas, as excreta are more likely to be flushed away by floodwaters. This result is consistent with the control of a case study conducted in Ecuador during the wet season (Bhavnani et al. 2014). These findings imply that while environmental factors in SFP areas may affect health, pathogen concentration in such areas is low, thus reducing the risk of possible pathogen contact. The sensitivity analysis performed by excluding only severe drought-prone areas showed different results from those of the main analysis. In Model I, the main analysis showed that the MFP area and its interaction with unimproved facilities were significantly associated with diarrhea prevalence; however, the sensitivity analysis showed no significant results for these variables. On the other hand, the LFP area showed a significant association with diarrhea prevalence. In Model II, the main analysis showed that the transferred sanitation type showed no significant association. In contrast, the sensitivity analysis showed a significant positive association with diarrhea prevalence. The differences in the results of the sensitivity analysis compared to those of the main analysis indicate that different factors control exposure to pathogens in high population density areas. For example, high population density areas have an increased possibility of human-to-human transmission of diseases, which may increase the susceptibility of vulnerable populations, such as children < 5 years of age, to diarrhea.

Model II showed that the LFP area was negatively associated with diarrhea prevalence compared to the NFP area, which implies that diarrhea risk may be worse in NFP areas than in flood-prone areas. Previous studies, on the other hand, mostly considered only flood-prone areas to examine the difference between the flood and non-flood periods. For example, during and after flood events cholera is highly prevalent; however, when humidity and temperature are low, diarrhea caused by rotavirus is more prevalent than cholera (Bray et al. 2019; Jagai et al. 2012; Schwartz et al. 2006). Joshi et al. (2011) evaluated the difference between the flood exposed and non-flood exposed groups to show that diarrhea prevalence among children was high in both the groups. Apart from the flood-prone areas, this study indicated that the NFP area should be focused on to identify other risk factors that can cause diarrhea among children < 5 years of age, and hence, further investigations are needed.

This study showed that shared sanitation facilities were positively associated with diarrhea prevalence in both Models I and II (Table S7), supporting the JMP ladder, which stated that shared sanitation facilities considered limited sanitation services rather than improved services (UNICEF and WHO 2019). Studies examining the Maputo sanitation (MapSan) trial have shown that improving shared sanitation facilities prevents human fecal contamination in target communities (Holcomb et al. 2020, 2021). The diarrhea prevalence rate among households with children < 5 years of age and shared sanitation facilities was significantly higher than that in households with non-shared facilities (Fuller et al. 2014). Furthermore, a previous study applied a mathematical model with fictitious pathogens and communities, demonstrating that high coverage of shared sanitation facilities increases the likelihood of contact with infected people and the risk of enteric infections (Just et al. 2018). Although numerous factors, such as socioeconomic status and caretaker education level, should also be considered (Heijnen et al. 2014), the result emphasizes that shared sanitation itself is a key risk factor resulting in adverse health outcomes.

Although the result of this study contradicts the JMP ladder criteria, some studies also showed mixed results regarding improved sanitation. For example, the Total Sanitation Campaign, one of the largest intervention projects conducted in India that aimed to end the practice of open defecation, revealed that installing sanitation facilities had no significant effect on reducing diarrhea prevalence despite decreasing the frequency of open defecation (Patil et al. 2014). Another intervention conducted in rural areas of Kenya and Bangladesh, also known as WaSH Benefits, showed different results. Contreras et al. (2022) showed that individual sanitation interventions (such as pit latrine installation, providing tools to clean child feces, and promoting behavioral change) reduced diarrhea prevalence, whereas Pickering et al. (2019) found that only integrated interventions involving drinking water, sanitation facilities, and nutrition effectively reduced the risk of helminth infection. A community-level study on hotspot districts of diarrhea prevalence in children reported increased rates in communities with low ratios of improved sanitation facilities (Azage et al. 2016). To summarize, community environmental and meteorological factors should also be considered.

Geological factors may be another key element. In this study, the interactions between flood risk level and sanitation type were significantly associated with diarrhea prevalence. In Model II, SFP areas had a significant positive association with diarrhea prevalence, but the interaction between the SFP area and diffused sanitation type had a significant negative association. This result may be explained by geological features in Bangladesh, which is located in the Bengal Delta, comprising the Ganges and Brahmaputra-Jamuna Deltas and the Sylhet Basin (Mukherjee et al. 2009; Umitsu 1987). As it is located in a coastal region and an alluvial lowland, the Bengal Delta experiences river flooding as well as flooding due to tidal surges (Umitsu 1987; Wesselink et al. 2015). As this alluvial floodplain contains sandy silt or sand, any infrastructure installed in this environment carries a high risk of inundation, destruction, or deterioration due to unstable sediments. In this environment, sanitation systems such as pit latrines, which collect and store fecal sludge in vaults for years, may not be safe, as fecal sludge can contaminate the surrounding aquifers in rural areas (Islam et al. 2016; Ravenscroft et al. 2017) or surface floods in urban areas (Jenkins et al. 2015). Unlike improved sanitation facilities, open defecation, or bucket latrines, the diffused sanitation type defined in this study may not contaminate the surrounding aquifer, because it does not collect sludge at a specific place, which in turn may avoid pathogen concentration. Similar results were observed for Model I—the MFP area had a significant positive association with diarrhea prevalence but a negative association when interacting with unimproved sanitation types. Further studies are needed; however, improved sanitation facilities did not exhibit a clear association with the reduction in diarrhea prevalence in flood-prone areas.

This study showed an increased risk of diarrhea in MFP and SFP areas, where prevalence was not reduced by improved or concentrated sanitation systems. However, this does not mean that unimproved sanitation, such as pit latrines without slabs or open defecation areas, presents a better option. A potential future system may involve fecal sludge emptying from storage vaults at specific intervals, followed by transfer and appropriate treatment. The effectiveness of implementing alternative sanitation systems, such as “constructed wetland system (CWS)” or “ecological sanitation toilets,” has been discussed, especially in low- and middle-income countries (Bydałek and Myszograj 2019; Jehawi et al. 2020; Langergraber and Muellegger 2005). In Bangladesh, the Reed Bed System, a CWS, has been proposed (Biswas 2014) and has recently been introduced to Rohingya camps (Kabir et al. 2020; Saeed et al. 2022). In this system, fecal sludge can be emptied, transferred, and treated appropriately and safely. Other advantages of these systems include their low cost and potential as a fertilizer source for agricultural use (Kabir et al. 2020). Examining and expanding alternative systems, including those mentioned previously, in flood-prone areas will contribute to the development of appropriate sanitation systems in Bangladesh.

This study has several limitations. Firstly, geodata for each cluster in all BDHS results were randomly displaced from their original location to maintain respondent confidentiality. Geodata could be displaced outside the district but could not cross divisional boundaries. The choropleth map was created at the district level; therefore, some clusters might have been displaced outside their original district. Secondly, because diarrhea episodes were based on caregivers’ self-reports, they were subject to recall bias. Thirdly, as the results of the sensitivity analysis, which included high population density areas, varied from those of the main analysis, there is possibility that in such areas people may be susceptible to diarrhea through pathways other than the influence of flood risk and sanitation level.

## Conclusions

Using aggregated BDHS data, this study showed the influence of flood risk and sanitation facilities on diarrhea prevalence among children < 5 years of age in Bangladesh. The MFP and SFP areas, affected by unimproved sanitation, showed negative associations with diarrhea prevalence. The findings indicated an increased risk of diarrhea in MFP and SFP areas. In addition, installing improved sanitation facilities may not directly contribute to diarrhea prevention. Geological and climatological features should be considered together to develop more appropriate sanitation technologies in regions with large floodplains, such as Bangladesh. In addition, future studies should focus on specific regions, such as SFP or NFP areas, to investigate latent factors that may affect diarrhea prevalence among children < 5 years of age.

## Supplementary Information

ESM 1 (DOCX 1853 kb)

## Data Availability

All data and materials associated with this study are presented within the manuscript.

## References

[CR1] Abbas HB, Routray JK (2014). Assessing factors affecting flood-induced public health risks in Kassala State of Sudan. Oper Res Health Care.

[CR2] Azage M, Kumie A, Worku A, Bagtzoglou AC (2016). Childhood diarrhea in high and low hotspot districts of Amhara Region, northwest Ethiopia: a multilevel modeling. J Heal Populat Nutrit.

[CR3] Bangladesh Agricultural Research Council (BARC), (2022) Government of the People’s Republic of Bangladesh. http://www.barc.gov.bd. Accessed 20 April 2022

[CR4] Bangladesh Water Development Board (2011) Annual flood report. http://ffwc.gov.bd/index.php/reports/annual-flood-reports. Accessed 13 March 2022

[CR5] Bangladesh Water Development Board (2014) Annual flood report. http://ffwc.gov.bd/index.php/reports/annual-flood-reports. Accessed 13 March 2022

[CR6] Bangladesh Water Development Board (2017) Annual flood report. http://ffwc.gov.bd/index.php/reports/annual-flood-reports. Accessed 13 March 2022

[CR7] Bangladesh Water Development Board (2018) Annual flood report. http://ffwc.gov.bd/index.php/reports/annual-flood-reports. Accessed 13 March 2022

[CR8] Beier H (2018). Situational peer effects on adolescents’ alcohol consumption: The moderating role of supervision, activity structure, and personal moral rules. Deviant Behav.

[CR9] Bhavnani D, Goldstick JE, Cevallos W, Trueba G, Eisenberg JNS (2014). Impact of rainfall on diarrheal disease risk associated with unimproved water and sanitation. Am J Trop Med Hyg.

[CR10] Biswas M, Roy DN, Tajmim A, Rajib SS, Hossain M, Farzana F, Yasmen N (2014). Prescription antibiotics for outpatients in Bangladesh: a cross-sectional health survey conducted in three cities. Ann Clin Microbiol Antimicrob.

[CR11] Brammer H (1990). Floods in Bangladesh: 1. Geographical background to the 1987 and 1988 floods. Geogr J.

[CR12] Bray AE, Ahmed S, Das SK, Khan SH, Chisti MJ, Ahmed T, Faruque ASG, Fuchs GJ (2019). Viral pathogen-specific clinical and demographic characteristics of children with moderate-to-severe diarrhea in Rural Bangladesh. Am J Trop Med Hyg.

[CR13] Burgert CR, Colston J, Roy T, Zachary B (2013) Geographic displacement procedure and georeferenced data release policy for the Demographic and Health Surveys. DHS Spatial Analysis Reports No. 7. ICF International, Calverton, MD. https://dhsprogram.com/pubs/pdf/SAR7/SAR7.pdf

[CR14] Bydałek F, Myszograj S (2019). Safe surface concept in vertical flow constructed wetland design to mitigate infection hazard. J Environ Sci Health A Tox Hazard Subst Environ Eng.

[CR15] Carlton EJ, Eisenberg JN, Goldstick J, Cevallos W, Trostle J, Levy K (2014). Heavy rainfall events and diarrhea incidence: the role of social and environmental factors. Am J Epidemiol.

[CR16] Cha S, Lee JE, Seo DS, Park BM, Mansiangi P, Hwang JS, Lee J (2017). Associations between household latrines and the prevalence of diarrhea in Idiofa, Democratic Republic of the Congo: A cross-sectional study. Am J Trop Med Hyg.

[CR17] Chowdhury F, Khan IA, Patel S, Siddiq AU, Saha NC, Khan AI, Saha A, Cravioto A, Clemens J, Qadri F, Ali M (2015). Diarrheal illness and healthcare seeking behavior among a population at high risk for diarrhea in Dhaka, Bangladesh. PLoS One.

[CR18] Chowdhury FR, Ibrahim QSU, Bari MS, Alam MMJ, Dunachie SJ, Rodriguez-Morales AJ, Patwary MI (2018). The association between temperature, rainfall and humidity with common climate-sensitive infectious diseases in Bangladesh. PLoS One.

[CR19] Borges Pedro JP, Oliveira CADS, de Lima SCRB, von Sperling M (2020). A review of sanitation technologies for flood-prone areas. J Water Sanit Hyg Dev.

[CR20] Chowdhury AMR, Bhuiya A, Chowdhury ME, Rasheed S, Hussain Z, Chen LC (2013). The Bangladesh paradox: exceptional health achievement despite economic poverty. The Lancet.

[CR21] Clasen T, Boisson S, Routray P, Torondel B, Bell M, Cumming O, Ensink J, Freeman M, Jenkins M, Odagiri M, Ray S, Sinha A, Suar M, Schmidt WP (2014). Effectiveness of a rural sanitation programme on diarrhoea, soil-transmitted helminth infection, and child malnutrition in Odisha, India: a cluster-randomised trial. Lancet Glob Health.

[CR22] Contreras JD, Islam M, Mertens A, Pickering AJ, Arnold BF, Benjamin-Chung J, Hubbard AE, Rahman M, Unicomb L, Luby SP, Colford JM, Ercumen A (2022). Evaluation of an on-site sanitation intervention against childhood diarrhea and acute respiratory infection 1 to 3.5 years after implementation: extended follow-up of a cluster-randomized controlled trial in rural Bangladesh. PLoS Med.

[CR23] Dagnew AB, Tewabe T, Miskir Y, Eshetu T, Kefelegn W, Zerihun K, Urgessa M, Teka T (2019). Prevalence of diarrhea and associated factors among under-five children in Bahir Dar city, Northwest Ethiopia, 2016: A cross-sectional study. BMC Infect Dis.

[CR24] Denslow SA, Edwards J, Horney J, Peña R, Wurzelmann D, Morgan D (2010). Improvements to water purification and sanitation infrastructure may reduce the diarrheal burden in a marginalized and flood prone population in remote Nicaragua. BMC Int Health Hum Rights.

[CR25] Dewan TH (2015). Societal impacts and vulnerability to floods in Bangladesh and Nepal. Weather Clim Extremes.

[CR26] Dey NC, Parvez M, Dey D, Saha R, Ghose L, Barua MK, Islam A, Chowdhury MR (2017). Microbial contamination of drinking water from risky tubewells situated in different hydrological regions of Bangladesh. Int J Hyg Environ Health.

[CR27] Dimitrova A, Muttarak R (2020) After the floods: differential impacts of rainfall anomalies on child stunting in India. Glob Environ Change 64. 10.1016/j.gloenvcha.2020.102130

[CR28] Dzwairo B, Hoko Z, Love D, Guzha E (2006). Assessment of the impacts of pit latrines on groundwater quality in rural areas: a case study from Marondera district, Zimbabwe. Phys Chem Earth Parts A B C.

[CR29] Fuller JA, Clasen T, Heijnen M, Eisenberg JN (2014). Shared sanitation and the prevalence of diarrhea in young children: evidence from 51 countries, 2001–2011. Am J Trop Med Hyg.

[CR30] Government of the People’s Republic of Bangladesh, Department of Public Health Engineering (DPHE) (2014) DPHE Publication #1, December 2014: Faecal Sludge Treatment Plant (Reed Bed System): A Technology of Faecal Sludge Management in Sub-Urban Regions of Bangladesh. https://itn.buet.ac.bd/web/resources/faecal-sludge-treatment-plant-reed-bed-system-a-technology-of-faecal-sludge-management-in-sub-urban-regions-of-bangladesh/. Accessed 15 September 2022

[CR31] Government of the People’s Republic of Bangladesh, Ministry of Health and Family Welfare (GOB-MHFW) (2019) Health Bulletin 2019. GOB-MHFW 2019. https://old.dghs.gov.bd/index.php/en/publications/health-bulletin/dghs-health-bulletin. Accessed 15 February 2023

[CR32] Hashimoto M, Suetsugi T, Ichikawa Y, Sunada K, Nishida K, Kondo N, Ishidaira H (2014). Assessing the relationship between inundation and diarrhoeal cases by flood simulations in low-income communities of Dhaka City, Bangladesh. Hydrol Res Lett.

[CR33] Hashizume M, Wagatsuma Y, Faruque ASG, Hayashi T, Hunter PR, Armstrong B, Sack DA (2008). Factors determining vulnerability to diarrhoea during and after severe floods in Bangladesh. J Water Health.

[CR34] Hashizume M, Armstrong B, Hajat S, Wagatsuma Y, Faruque ASG, Hayashi T, Sack DA (2008). The effect of rainfall on the incidence of cholera in Bangladesh. Epidemiology.

[CR35] Heijnen M, Rosa G, Fuller J, Eisenberg JNS, Clasen T (2014). The geographic and demographic scope of shared sanitation: an analysis of national survey data from low- and middle-income countries. Trop Med Int Health.

[CR36] Holcomb DA, Knee J, Capone D, Sumner T, Adriano Z, Nalá R, Cumming O, Brown J, Stewart JR (2021). Impacts of an urban sanitation intervention on fecal indicators and the prevalence of human fecal contamination in Mozambique. Environ Sci Technol.

[CR37] Holcomb DA, Knee J, Sumner T, Adriano Z, de Bruijn E, Nalá R, Cumming O, Brown J, Stewart JR (2020). Human fecal contamination of water, soil, and surfaces in households sharing poor-quality sanitation facilities in Maputo. Mozambique. Int J Hyg Environ Health.

[CR38] [dataset] The Humanitarian Data Exchange. (2021). Bangladesh – Hazards (Drought risk, Earthquake risk, Flood risk and River erosion risk). https://data.humdata.org/dataset/bangladesh-hazards#. Accessed 20 April 2022

[CR39] Islam MS, Mahmud ZH, Islam MS, Saha GC, Zahid A, Ali AZ, Hassan MQ, Islam K, Jahan H, Hossain Y, Hasan MM, Cairncross S, Carter R, Luby SP, Cravioto A, Endtz HP, Faruque SM, Clemens JD (2016). Safe distances between groundwater-based water wells and pit latrines at different hydrogeological conditions in the Ganges Atrai floodplains of Bangladesh. J Health Popul Nutr.

[CR40] Jagai JS, Sarkar R, Castronovo D, Kattula D, McEntee J, Ward H, Kang G, Naumova EN (2012). Seasonality of rotavirus in South Asia: A meta-analysis approach assessing associations with temperature, precipitation, and vegetation index. PLoS ONE.

[CR41] Jehawi OH, Abdullah SRS, Kurniawan SB, Ismail N, Idris M, Al Sbani NH, Muhamad MH, Hasan HA (2020). Performance of pilot Hybrid Reed Bed constructed wetland with aeration system on nutrient removal for domestic wastewater treatment. Environ Technol Innov. Muhamad, NH. Highways Agency.

[CR42] Jenkins MW, Cumming O, Cairncross S (2015). Pit latrine emptying behavior and demand for sanitation services in Dar es Salaam, Tanzania. Int J Environ Res Public Health.

[CR43] Joshi PC, Kaushal S, Aribam BS, Khattri P, D’Aoust O, Singh MM, Marx M, Guha-Sapir D (2011). Recurrent floods and prevalence of diarrhea among under five children: observations from Bahraich District, Uttar Pradesh, India. Glob Health Action.

[CR44] Just MR, Carden SW, Li S, Baker KK, Gambhir M, Fung ICH (2018). The impact of shared sanitation facilities on diarrheal diseases with and without an environmental reservoir: A modeling study. Pathog Glob Health.

[CR45] Kabir MI, Hoque MA, Banik BK (2020). Performance of a reed bed system for faecal wastewater treatment: case study. Water Pract Technol.

[CR46] Kamal MM, Hasan MM, Davey R (2015). Determinants of childhood morbidity in Bangladesh: evidence from the demographic and health survey 2011. BMJ Open.

[CR47] Knee J, Sumner T, Adriano Z, Berendes D, de Bruijn E, Schmidt WP, Nalá R, Cumming O, Brown J (2018). Risk factors for childhood enteric infection in urban Maputo, Mozambique: A cross-sectional study. PLoS Negl Trop Dis.

[CR48] Langergraber G, Muellegger E (2005). Ecological Sanitation—a way to solve global sanitation problems?. Environ Int.

[CR49] Levy K, Woster AP, Goldstein RS, Carlton EJ (2016). Untangling the impacts of climate change on waterborne diseases: a systematic review of relationships between diarrheal diseases and temperature, rainfall, flooding, and drought. Environ Sci Technol.

[CR50] Local Burden of Disease WaSH Collaborators (2020). Mapping geographical inequalities in access to drinking water and sanitation facilities in low-income and middle-income countries 2000–17. Lancet Glob Health.

[CR51] Luby SP, Rahman M, Arnold BF, Unicomb L, Ashraf S, Winch PJ, Stewart CP, Begum F, Hussain F, Benjamin-Chung J, Leontsini E, Naser AM, Parvez SM, Hubbard AE, Lin A, Nizame FA, Jannat K, Ercumen A, Ram PK, Das KK, Abedin J, Clasen TF, Dewey KG, Fernald LC, Null C, Ahmed T, Colford JM (2018). Effects of water quality, sanitation, handwashing, and nutritional interventions on diarrhoea and child growth in rural Bangladesh: a cluster randomised controlled trial. Lancet Glob Health.

[CR52] Manga M, Kolsky P, Rosenboom JW, Ramalingam S, Sriramajayam L, Bartram J, Stewart J (2022). Public health performance of sanitation technologies in Tamil Nadu, India: Initial perspectives based on *E. coli* release. Int J Hyg Environ Health.

[CR53] Milojevic A, Armstrong B, Hashizume M, McAllister K, Faruque A, Yunus M, Kim Streatfield PK, Moji K, Wilkinson P (2012). Health effects of flooding in rural Bangladesh. Epidemiology.

[CR54] Mood C (2010). Logistic regression: why we cannot do what we think we can do, and what we can do about it. Eur Sociol Rev.

[CR55] Mukherjee A, Fryar AE, Thomas WA (2009). Geologic, geomorphic and hydrologic framework and evolution of the Bengal basin, India and Bangladesh. J Asian Earth Sci.

[CR56] Null C, Stewart CP, Pickering AJ, Dentz HN, Arnold BF, Arnold CD, Benjamin-Chung J, Clasen T, Dewey KG, Fernald LCH, Hubbard AE, Kariger P, Lin A, Luby SP, Mertens A, Njenga SM, Nyambane G, Ram PK, Colford JM (2018). Effects of water quality, sanitation, handwashing, and nutritional interventions on diarrhoea and child growth in rural Kenya: a cluster-randomised controlled trial. Lancet Glob Health.

[CR57] Omotayo AO, Olagunju KO, Omotoso AB, Ogunniyi AI, Otekunrin OA, Daud AS (2021). Clean water, sanitation and under-five children diarrhea incidence: Empirical evidence from the South Africa’s General Household Survey. Environ Sci Pollut Res.

[CR58] Patil SR, Arnold BF, Salvatore AL, Briceno B, Ganguly S, Colford JM, Gertler PJ (2014). The effect of India’s total sanitation campaign on defecation behaviors and child health in rural Madhya Pradesh: A cluster randomized controlled trial. PLoS Med.

[CR59] Peal A, Evans B, Blackett I, Hawkins P, Heymans C (2015) A review of fecal sludge management in 12 cities. Water and Sanitation Program (WSP), World Bank Final Report. https://www.susana.org/_resources/documents/default/3-2212-7-1435304068.pdf. Accessed 10 Month 2023

[CR60] Pickering AJ, Njenga SM, Steinbaum L, Swarthout J, Lin A, Arnold BF, Stewart CP, Dentz HN, Mureithi M, Chieng B, Wolfe M, Mahoney R, Kihara J, Byrd K, Rao G, Meerkerk T, Cheruiyot P, Papaiakovou M, Pilotte N, Williams SA, Colford JM, Null C (2019). Effects of single and integrated water, sanitation, handwashing, and nutrition interventions on child soil-transmitted helminth and *Giardia* infections: A cluster-randomized controlled trial in rural Kenya. PLoS Med.

[CR61] R Core Team (2021) R: A Language and Environment for Statistical Computing. https://www.R-project.org/. R Foundation for Statistical Computing, Vienna, Austria. Accessed 27 April 2022.

[CR62] Ravenscroft P, Mahmud ZH, Islam MS, Hossain AKMZ, Zahid A, Saha GC, Zulfiquar Ali AHM, Islam K, Cairncross S, Clemens JD, Islam MS (2017). The public health significance of latrines discharging to groundwater used for drinking. Water Res.

[CR63] Saeed T, Majed N, Kumar Yadav A, Hasan A, Jihad Miah M (2022). Constructed wetlands for drained wastewater treatment and sludge stabilization: role of plants, microbial fuel cell and earthworm assistance. Chem Eng J.

[CR64] Schwartz BS, Harris JB, Khan AI, Larocque RC, Sack DA, Malek MA, Faruque ASJ, Qadri F, Calderwood SB, Luby SP, Ryan ET (2006). Diarrheal epidemics in Dhaka, Bangladesh, during three consecutive floods: 1988, 1998, and 2004. Am J Trop Med Hyg.

[CR65] Shamsudduha M, Uddin A (2007). Quaternary shoreline shifting and hydrogeologic influence on the distribution of groundwater arsenic in aquifers of the Bengal basin. J Asian Earth Sci.

[CR66] Sinha A, Nagel CL, Schmidt WP, Torondel B, Boisson S, Routray P, Clasen TF (2017). Assessing patterns and determinants of latrine use in rural settings: A longitudinal study in Odisha, India. Int J Hyg Environ Health.

[CR67] Sinmegn Mihrete T, Asres Alemie G, Shimeka Teferra A (2014). Determinants of childhood diarrhea among underfive children in Benishangul Gumuz regional state. North West Ethiopia. BMC Pediatr.

[CR68] Umitsu M (1993). Late Quaternary sedimentary environments and landforms in the Ganges Delta. Sedimentary Geology.

[CR69] Umitsu M (1987). Late Quaternary sedimentary environment and landform evolution in the Bengal lowland. Geogr Rev Jpn Ser B.

[CR70] United Nations Children’s Fund (UNICEF) and World Health Organization (WHO) (2019) Progress on household drinking water, sanitation and hygiene 2000–2017: special focus on inequalities. https://data.unicef.org/resources/progress-drinking-water-sanitation-hygiene-2019/#:~:text=The%20WHO%2FUNICEF%20JMP%20report%2C%20Progress%20on%20household%20drinking,populations%20most%20at%20risk%20of%20being%20left%20behind. Accessed 15 September 2022.

[CR71] Wesselink A, Warner J, Syed MA, Chan F, Tran DD, Huq HH, Huthoff F, Thuy NL, Pinter N, Staveren MV, Wester P, Zegwaard A (2015) Trends in flood risk management in deltas around the world: are we going “soft”? Int J Water Gov 3:25–46. https://www.bing.com/ck/a?!&&p=8a5d00bc34e38996JmltdHM9MTY5MDY3NTIwMCZpZ3VpZD0yMDc5MjM4NS0zN2M0LTYxNmEtMGQ4Yi0zMGRiMzYyZTYwZTkmaW5zaWQ9NTE4OQ&ptn=3&hsh=3&fclid=20792385-37c4-616a-0d8b-30db362e60e9&psq=DOI%3a+10.7564%2f15-IJWG90&u=a1aHR0cHM6Ly93d3cucmVzZWFyY2hnYXRlLm5ldC9wdWJsaWNhdGlvbi8yODk1Mzg5NDhfVHJlbmRzX2luX2Zsb29kX3Jpc2tfbWFuYWdlbWVudF9pbl9kZWx0YXNfYXJvdW5kX3RoZV93b3JsZF9BcmVfd2VfZ29pbmdfJ3NvZnQn&ntb=1. Accessed 10 Oct 2022

[CR72] Yin H, Islam MS, Ju M (2021). Urban river pollution in the densely populated city of Dhaka, Bangladesh: big picture and rehabilitation experience from other developing countries. J Cleaner Prod.

[CR73] Zhang N, Song D, Zhang J, Liao W, Miao K, Zhong S, Lin S, Hajat S, Yang L, Huang C (2019). The impact of the 2016 flood event in Anhui Province, China on infectious diarrhea disease: an interrupted time-series study. Environ Int.

