# Supplementary material for: Influence of sanitation facilities on diarrhea prevalence among children aged below 5 years in flood-prone areas of Bangladesh: a multilevel analysis
Source: Environ Sci Pollut Res Int. 2023 Aug 21;30(43):97925–35. doi: 10.1007/s11356-023-29373-0 (PMC10495509; doi:10.1007/s11356-023-29373-0)
Supplement: Supplementary file 1 — (DOCX 1853 kb) [file 11356_2023_29373_MOESM1_ESM.docx]

**Supplementary material for**

**Influence of sanitation facilities on diarrhea prevalence among children aged below 5 years in flood-prone areas of Bangladesh: A multilevel analysis**

Contents

Table S1: Flood prone area used in this study

Table S2: Sanitation facilities based on Containment type coded for Model I

Table S3: Sanitation facilities based on Excreta flow coded for Model II

Table S4: Sanitation facilities coded for analysis (comparison)

Table S5: Four types of floods in Bangladesh

Table S6: Independent variables used in this study

Table S7: Multilevel linear probability models (Model I and Model II)

Table S8: Sensitivity analysis including high population density areas

Fig. S1: Flood prone areas map (four levels)

Fig. S2: Severe drought risk area map (original from BARC)

Fig. S3: The diarrhea prevalence of children under five in 2011

Fig. S4: The diarrhea prevalence of children under five in 2014

Fig. S5: The diarrhea prevalence of children under five in 2017-2018

Fig. S6: The diarrhea prevalence of children under five, sum of DHS2011, 2014 and 2017-2018

Table S1: Flood prone area used in this study

| Flood | Mechanism | Area:km^2^ (%) |
| --- | --- | --- |
| Severe | Occurs 5 times or more in 10 years with considerable loss/damage of standing crops | 23,621 (16.0) |
| Moderate | Occurs 3-4 times in 10 years with significant loss/damage of standing crops | 33,955 (23.0) |
| Low | Occurs at least twice in 10 years with loss/damage of standing crops | 34,250 (23.2) |
| Not flood prone | None in 10 years | 55,804 (37.8) |

(Made by author, Source: BARC)

Table S2: Sanitation facilities based on Containment type coded for Model I

| Containment type | Sanitation facilities |
| --- | --- |
| Improved | flush to piped sewer system |
|  | flush to septic tank |
|  | flush to pit latrine |
|  | ventilated improved pit latrine (VIP) |
|  | Pit latrine with slab |
|  | Composting toilet |
| Unimproved | flush to somewhere else |
|  | flush, don't know where |
|  | pit latrine without slab/open pit |
|  | bucket toilet |
|  | hanging toilet/latrine |
|  | no facility/bush/field |

(made by author, source: DHS and UNICEF/WHO 2019)

Table S3: Sanitation facilities based on Excreta flow coded for Model II

| Excreta flow | Sanitation facilities |
| --- | --- |
| Concentrated | pit latrine with slab |
|  | pit latrine without slab/open pit |
|  | ventilated improved pit latrine (VIP) |
|  | Flush to pit latrine |
|  | Flush to somewhere else |
|  | Flush, don’t know where |
|  | Flush to septic tank |
| Diffused | no facility/bush/field |
|  | Bucket toilet |
|  | Composting toilet |
|  | Hanging toilet / latrine |
| transferred | flush to piped sewer system |

(made by author, source: DHS and UNICEF/WHO 2019)

Table S4: Sanitation facilities coded for analysis (comparison)

| kinds of sanitation facilities | Model I (containment type) | Model II (excreta flow) |
| --- | --- | --- |
| flush to piped sewer system | Improved | Transferred |
| flush to septic tank | Improved | Concentrated |
| flush to pit latrine | Improved | Concentrated |
| flush to somewhere else | Not improved | Concentrated |
| flush, don't know where | Not improved | Concentrated |
| ventilated improved pit latrine (vip) | Improved | Concentrated |
| pit latrine with slab | Improved | Concentrated |
| pit latrine without slab/open pit | Not improved | Concentrated |
| no facility/bush/field | Not improved | Diffused |
| composting toilet | Improved | Diffused |
| bucket toilet | Not improved | Diffused |
| hanging toilet/latrine | Not improved | Diffused |

(made by author, source: DHS and UNICEF/WHO 2019)

Table S5: Four types of floods in Bangladesh

| Flood | Mechanism | season | Region | area: Mha (%) |
| --- | --- | --- | --- | --- |
| River Flooding | Overtopping of the riverbanks in monsoon season | monsoon season  (June to September or October) | The catchment of Ganges, Brahmaputra and Meghna rivers | 5.62 (40.40) |
| Flash Flooding | Heavy run-off from the higher sites due to torrential rainfall in early monsoon season | early monsoon season  (April to May and September to November | Eastern and northern area | 1.47 (10.57) |
| Tidal Surge | generated mainly by wind stress and falling atmospheric pressure that produce a rise in water level in coastal area (Karim and Mimura, 2008) | early or late monsoon season | Southern coastal area | 1.18 (8.48) |
| Not flood prone |  |  |  | 5.64 (40.55) |

(Made by author, Source: BARC; Karim and Mimura, 2008; BWDB)

Table S6: Independent variables used in this study

| variables |  | measurement of variables |
| --- | --- | --- |
| Outcome variable | | |
|  | Diarrhea incidence in the past two weeks | Categorized into “yes”, “no” |
| Explanatory variables | | |
| [Individual Level] | | |
|  | Child age | Categorized into “0-11month”, “12-23month”, “24-35month”, “36-47month”, “48-59month” |
|  | Sex of child | Categorized into “male” and “female” |
|  | JMP ladder only for Model I | Categorized into “improved” and “not improved” |
|  | Sanitation type only for Model II | Categorized into “transferred”, “concentrated” and “diffused” |
|  | Toilet shared or individual | Categorized into “shared“, “individual“ and “no facilities“ |
|  | Type of water | Categorized into “improved” and “improved” |
|  | Mother’s age | Mother’s age at the survey |
|  | Mother’s education | Categorized into “high school”, “secondary school”, “primary school” and “no education” |
|  | Wealth index | Categorized into “richest”, “richer”, “middle”, “poorer” and “poorest” |
|  | Religion | Categorized into “Muslim” and “others” |
| [Community Level] | | |
|  | Residential area | Categorized into “rural”, and “urban” |
|  | Flood prone area | Categorized into “Severely flood-prone (SFP)”, “MFP Moderately flood-prone (MFP)”, “Low flood-prone (LFP)” and “Not flood prone (NFP)” |
|  | Population of under-five ages | Each survey year refers data obtained in every five years, for example DHS2011 refers 2010, DHS2014 refers 2015, and DHS2017-2018 refers 2020 |

(made by author, source: DHS)

Table S7: Multilevel linear probability models (Models I and II)

|  | | Model I: Containment type | | | | Model II: Excreta flow | | | |
| --- | --- | --- | --- | --- | --- | --- | --- | --- | --- |
|  | | without interaction | | with interaction | | without interaction | | with interaction | |
|  | | β | 95% CI | β | 95% CI | β | 95% CI | β | 95% CI |
| **Containment type (Model I)** | |  |  |  |  |  |  |  |  |
| Improved | | (reference) |  | (reference) |  |  |  |  |  |
| Unimproved | | 0.003 | (–0.004, 0.011) | 0.015 | (–0.0004, 0.026) |  |  |  |  |
| **Excreta flow (Model II)** | |  |  |  |  |  |  |  |  |
| Concentrated | |  |  |  |  | (reference) |  | (reference) |  |
| Transferred | |  |  |  |  | 0.023 | (–0.017, 0.064) | 0.030 | (–0.184, 0.079) |
| Diffused | |  |  |  |  | 0.004 | (–0.012, 0.019) | 0.027 | (0.004, 0.053) |
| **Flood type area** | |  |  |  |  |  |  |  |  |
| Not flood-prone (NFP) | | (reference) |  | (reference) |  | (reference) |  | (reference) |  |
| Low (LFP) | | –0.013 | (–0.023, –0.003) | –0.009 | (–0.023, 0.003) | –0.013 | (–0.023, –0.003) | –0.113 | (–0.024, –0.001) |
| Moderate (MFP) | | 0.003 | (–0.005, 0.012) | 0.012 | (0.001, 0.024) | 0.003 | (–0.005, 0.012) | 0.005 | (–0.004, 0.014) |
| Severe (SFP) | | 0.015 | (0.001, 0.027) | 0.024 | (–0.003, 0.030) | 0.015 | (0.002, 0.028) | 0.022 | (0.008, 0.035) |
| **Interaction (Model I)** | |  |  |  |  |  |  |  |  |
| Unimproved * LFP area | |  |  | –0.009 | (–0.029, 0.009) |  |  |  |  |
| Unimproved * MFP area | |  |  | –0.022 | (–0.040, –0.004) |  |  |  |  |
| Unimproved * SFP area | |  |  | 0.003 | (–0.019, 0.028) |  |  |  |  |
| **Interaction (Model II)** | |  |  |  |  |  |  |  |  |
| Transferred * LFP area | |  |  |  |  |  |  | –0.025 | (–0.121, 0.083) |
| Diffused * LFP area | |  |  |  |  |  |  | –0.021 | (–0.057, 0.012) |
| Transferred * MFP area | |  |  |  |  |  |  | –0.066 | (–0.224, 0.099) |
| Diffused * MFP area | |  |  |  |  |  |  | –0.021 | (–0.055, 0.012) |
| Transferred * SFP area | |  |  |  |  |  |  | 0.037 | (–0.145, 0.210) |
| Diffused * SFP area | |  |  |  |  |  |  | –0.060 | (–0.095, –0.023) |
| **Child age** |  | |  |  |  |  |  |  |  |
| 0~11 months | (reference) | |  | (reference) |  | (reference) |  | (reference) |  |
| 12~23 months | 0.025 | | (0.014, 0.101) | 0.025 | (0.014, 0.037) | 0.025 | (0.013, 0.037) | 0.025 | (0.014, 0.037) |
| 24~35 months | –0.012 | | (–0.024, –0.001) | –0.012 | (–0.024, –0.001) | –0.012 | (–0.023, –0.001) | –0.012 | (–0.023, –0.001) |
| 36~47 months | –0.025 | | (–0.036, –0.014) | –0.025 | (–0.036, –0.014) | –0.025 | (–0.036, –0.014) | –0.025 | (–0.036, –0.014) |
| 48~59 months | –0.030 | | (–0.042, –0.018) | –0.030 | (–0.041, –0.019) | –0.030 | (–0.042, –0.019) | –0.031 | (–0.042, –0.019) |
| **Child sex** |  | |  |  |  |  |  |  |  |
| Female | (reference) | |  | (reference) |  | (reference) |  | (reference) |  |
| Male | 0.009 | | (0.002, 0.016) | 0.009 | (0.002, 0.016) | 0.009 | (0.001, 0.017) | 0.009 | (0.002, 0.016) |
| **Mother’s age** | –0.0007 | | (-0.001, -0.0001) | –0.0007 | (–0.001, –0.0001) | –0.0007 | (–0.001, 0.0001) | –0.0007 | (–0.001, –0.0001) |
| **Mother’s education** |  | |  |  |  |  |  |  |  |
| High school | (reference) | |  | (reference) |  | (reference) |  | (reference) |  |
| Secondary | –0.002 | | (–0.016, 0.013) | –0.002 | (–0.017, 0.010) | –0.018 | (–0.015, 0.010) | –0.002 | (–0.015, 0.011) |
| Primary | 0.011 | | (–0.004, 0.026) | 0.010 | (–0.004, 0.026) | 0.011 | (–0.002, 0.025) | 0.011 | (–0.003, 0.027) |
| No education | 0.005 | | (–0.012, 0.022) | 0.005 | (–0.011, 0.022) | 0.006 | (–0.009, 0.021) | 0.005 | (–0.011, 0.022) |
| **Wealth index** |  | |  |  |  |  |  |  |  |
| Middle | (reference) | |  | (reference) |  | (reference) |  |  |  |
| Poorest | –0.009 | | (–0.021, 0.0008) | –0.009 | (–0.021, 0.002) | –0.009 | (–0.018, 0.002) | –0.008 | (–0.020, 0.002) |
| Poorer | –0.003 | | (–0.014, 0.007) | –0.003 | (–0.013, 0.008) | –0.028 | (–0.014, 0.010) | –0.003 | (–0.014, 0.008) |
| Richer | –0.015 | | (–0.026, –0.002) | –0.015 | (–0.027, –0.002) | –0.015 | (–0.025, –0.003) | –0.015 | (–0.027, –0.003) |
| Richest | 0.0003 | | (–0.013, 0.015) | 0.0004 | (–0.011, 0.022) | –0.001 | (–0.013, 0.012) | –0.0008 | (–0.014, 0.013) |
| **Religion** |  | |  |  |  |  |  |  |  |
| Islam | (reference) | |  | (reference) |  | (reference) |  | (reference) |  |
| Other | –0.011 | | (–0.022, 0.002) | –0.011 | (–0.024, 0.001) | –0.011 | (–0.024, 0.001) | –0.012 | (–0.025, 0.0005) |
| **Residential area** |  | |  |  |  |  |  |  |  |
| Rural | (reference) | |  | (reference) |  | (reference) |  | (reference) |  |
| Urban | 0.0007 | | (–0.008, 0.009) | 0.003 | (–0.008, 0.010) | 0.0006 | (–0.008, 0.010) | 0.0007 | (–0.009, 0.010) |
| **Population density^1)^** | 0.0006 | | (–0.016, 0.017) | 0.001 | (–0.016, 0.016) | –0.0005 | (–0.017, 0.017) | –0.0007 | (–0.016, 0.016) |
| **Type of water** |  | |  |  |  |  |  |  |  |
| Improved | (reference) | |  | (reference) |  | (reference) |  | (reference) |  |
| Unimproved | 0.003 | | (–0.004, 0.012) | –0.015 | (–0.039, 0.006) | –0.016 | (–0.039, 0.007) | –0.017 | (–0.043, 0.004) |
| **Toilet shared / private** |  | |  |  |  |  |  |  |  |
| Private | (reference) | |  | (reference) |  | (reference) |  | (reference) |  |
| Shared | 0.009 | | (0.0005, 0.016) | 0.009 | (0.002, 0.017) | 0.009 | (0.001, 0.016) | 0.009 | (0.0009, 0.016) |
| No facilities/bush | 0.008 | | (–0.009, 0.025) | 0.007 | (-0.011, 0.028) | 0.005 | (–0.017, 0.027) | 0.0001 | (–0.023, 0.023) |

1) scaled, 2) sani-: sanitation type

Dependent variable: diarrhea = yes, Model I: multilevel linear probability model of Containment type, Model II: multilevel linear probability model of Excreta flow

β: coefficient, CI: Confidence Interval

Table S8: Sensitivity analysis including high population density areas

|  | Model I: Containment type | | | | Model II: Excreta flow | | | |
| --- | --- | --- | --- | --- | --- | --- | --- | --- |
|  | without interaction | | with interaction | | without interaction | | with interaction | |
|  | β | 95% CI | β | 95% CI | β | 95% CI | β | 95% CI |
| **Containment type (Model I)** |  |  |  |  |  |  |  |  |
| Improved | (reference) |  | (reference) |  |  |  |  |  |
| Unimproved | 0.001 | (-0.006, 0.009) | 0.005 | (-0.006, 0.017) |  |  |  |  |
| **Excreta flow (Model II)** |  |  |  |  |  |  |  |  |
| Concentrated |  |  |  |  | (reference) |  | (reference) |  |
| Transferred |  |  |  |  | 0.034 | (0.008, 0.060) | 0.043 | (0.014, 0.071) |
| Diffused |  |  |  |  | 0.006 | (-0.011, 0.032) | 0.030 | (0.004, 0.055) |
| **Flood type area** |  |  |  |  |  |  |  |  |
| Not flood-prone (NFP) | (reference) |  | (reference) |  | (reference) |  | (reference) |  |
| Low (LFP) | -0.014 | (-0.024, -0.004) | -0.013 | (-0.026, -0.0005) | -0.014 | (-0.024, -0.045) | -0.011 | (-0.022, -0.001) |
| Moderate (MFP) | 0.002 | (-0.007, 0.011) | 0.008 | (-0.003, 0.020) | 0.002 | (-0.007, 0.011) | 0.004 | (-0.004, 0.014) |
| Severe (SFP) | 0.014 | (0.001, 0.027) | 0.008 | (-0.008, 0.025) | 0.014 | (0.001, 0.027) | 0.021 | (0.008, 0.034) |
| **Interaction (Model I)** |  |  |  |  |  |  |  |  |
| Unimproved * LFP area |  |  | -0.002 | (-0.021, 0.016) |  |  |  |  |
| Unimproved * MFP area |  |  | -0.015 | (-0.032, 0.002) |  |  |  |  |
| Unimproved * SFP area |  |  | 0.011 | (-0.012, 0.034) |  |  |  |  |
| **Interaction (Model II)** |  |  |  |  |  |  |  |  |
| Transferred * LFP area |  |  |  |  |  |  | -0.050 | (-0.011, 0.019) |
| Diffused * LFP area |  |  |  |  |  |  | -0.023 | (-0.057, 0.010) |
| Transferred * MFP area |  |  |  |  |  |  | -0.078 | (-0.208, 0.051) |
| Diffused * MFP area |  |  |  |  |  |  | -0.022 | (-0.053, 0.009) |
| Transferred * SFP area |  |  |  |  |  |  | 0.026 | (-0.136, 0.189) |
| Diffused * SFP area |  |  |  |  |  |  | -0.061 | (-0.099, -0.023) |


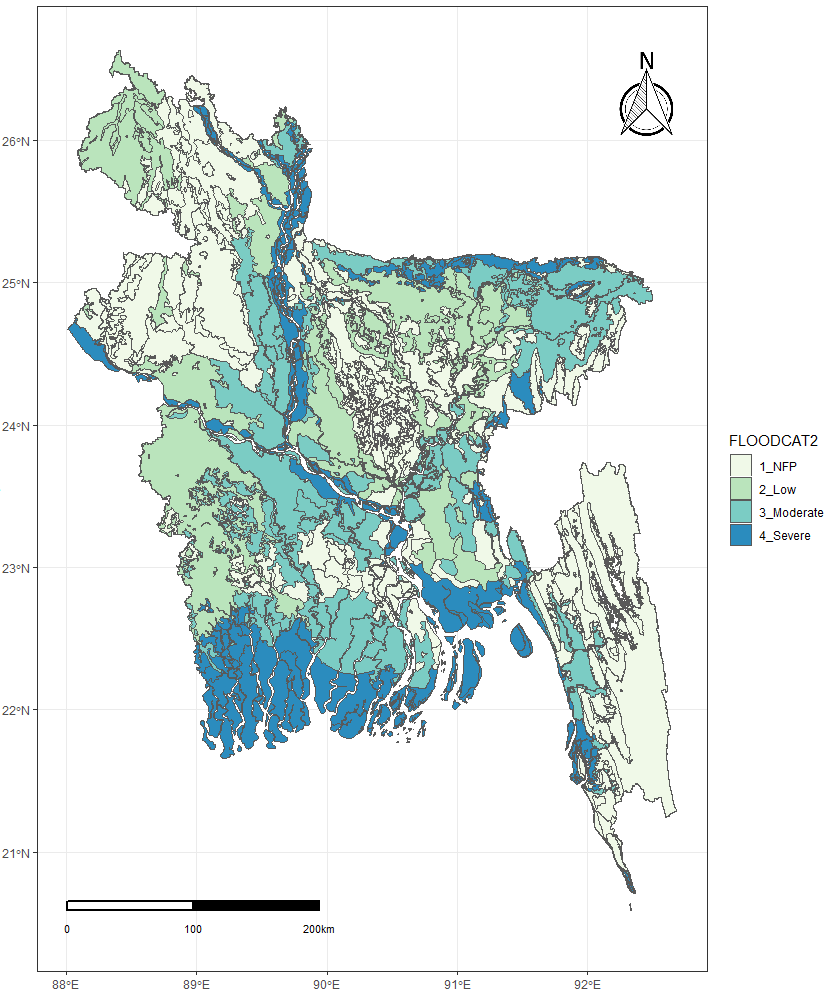


Not flood prone

Low

Moderate

Severe

Flood risk

Fig. S1: Flood prone area map (made by author, source: BARC)


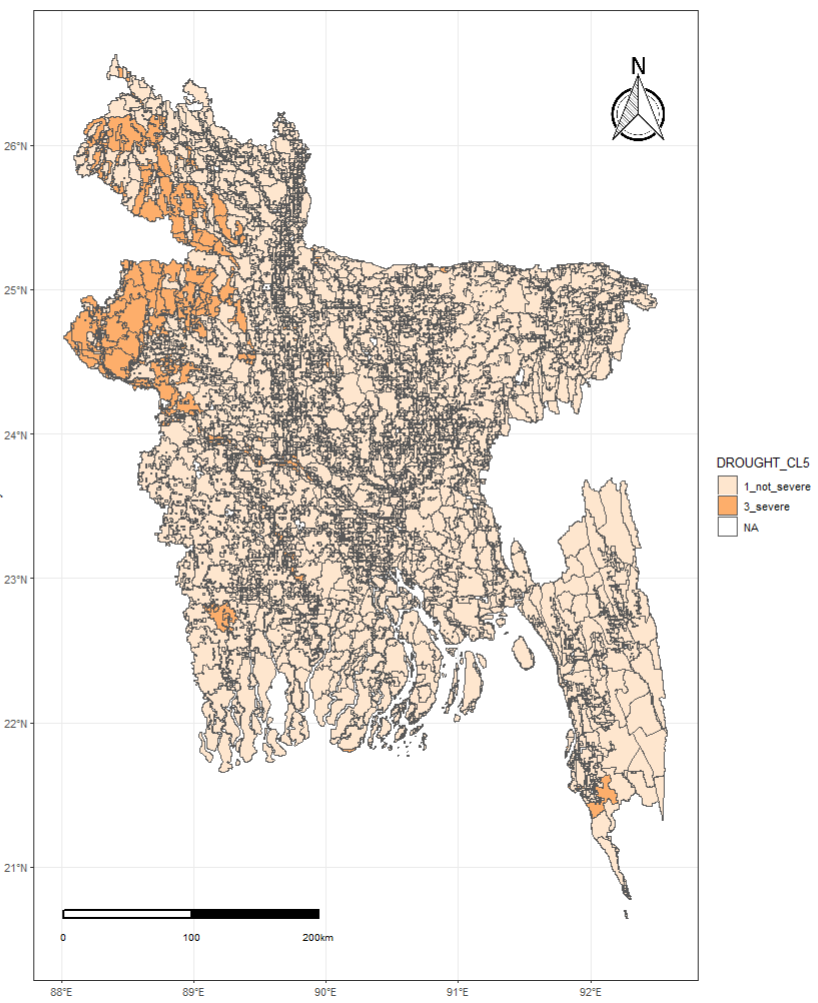


Not severe

Severe

No data

Drought risk area

Fig. S2: Severe drought areas of Bangladesh (Source: BARC)


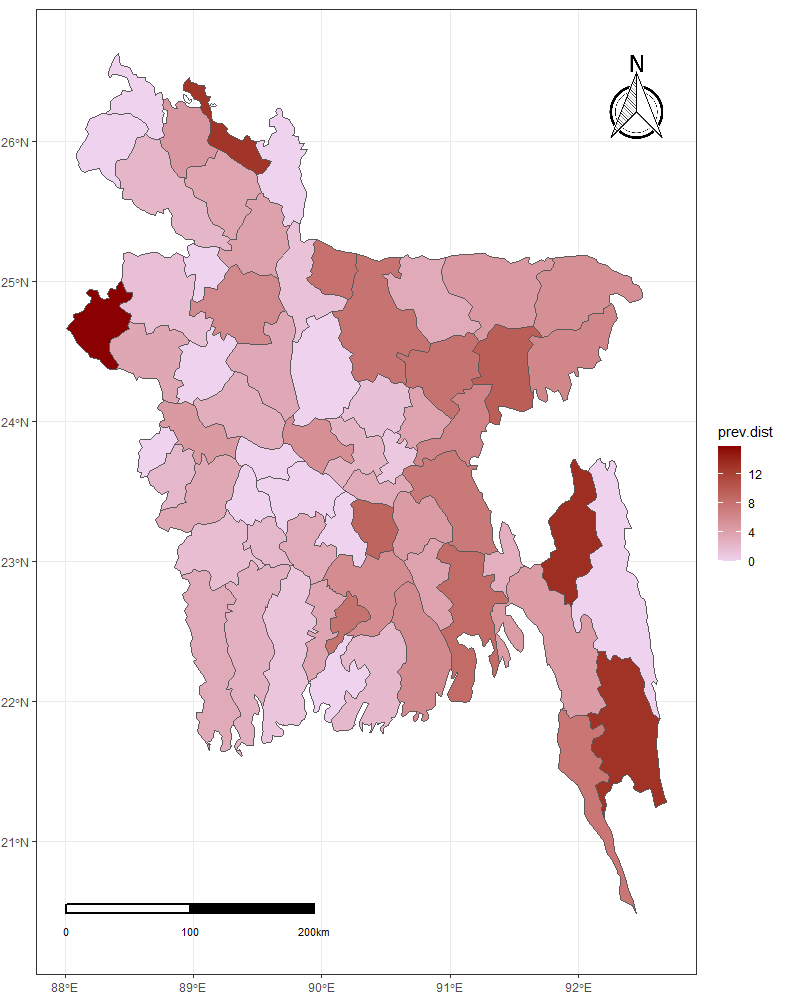


Prevalence

of diarrhea (%)

Fig. S3: The diarrhea prevalence of children under five in 2011

(made by author, Source: BDHS and BARC)


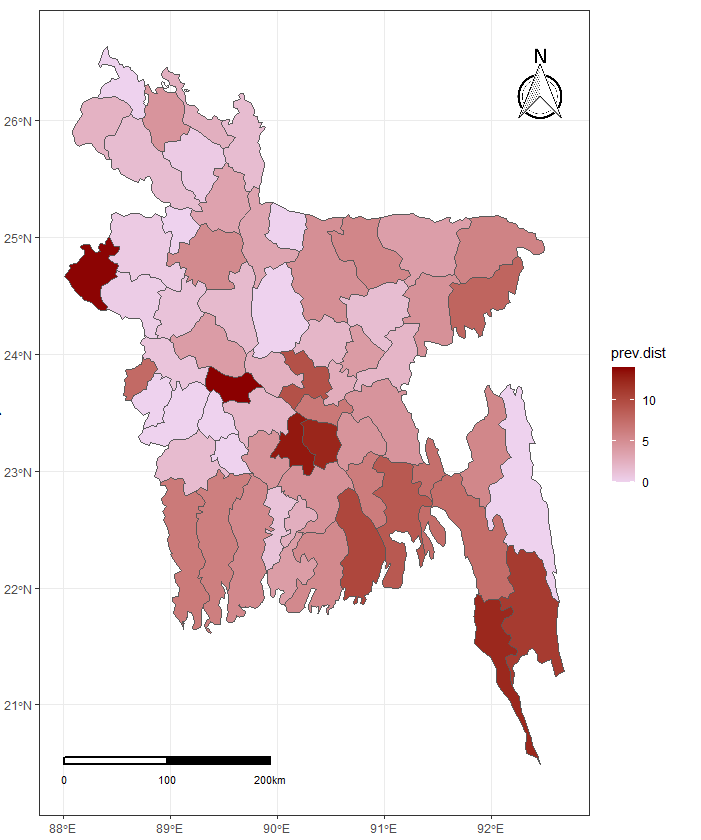


Prevalence

of diarrhea (%)

Fig. S4: The diarrhea prevalence of children under five in 2014

(made by author, Source: BDHS and BARC)


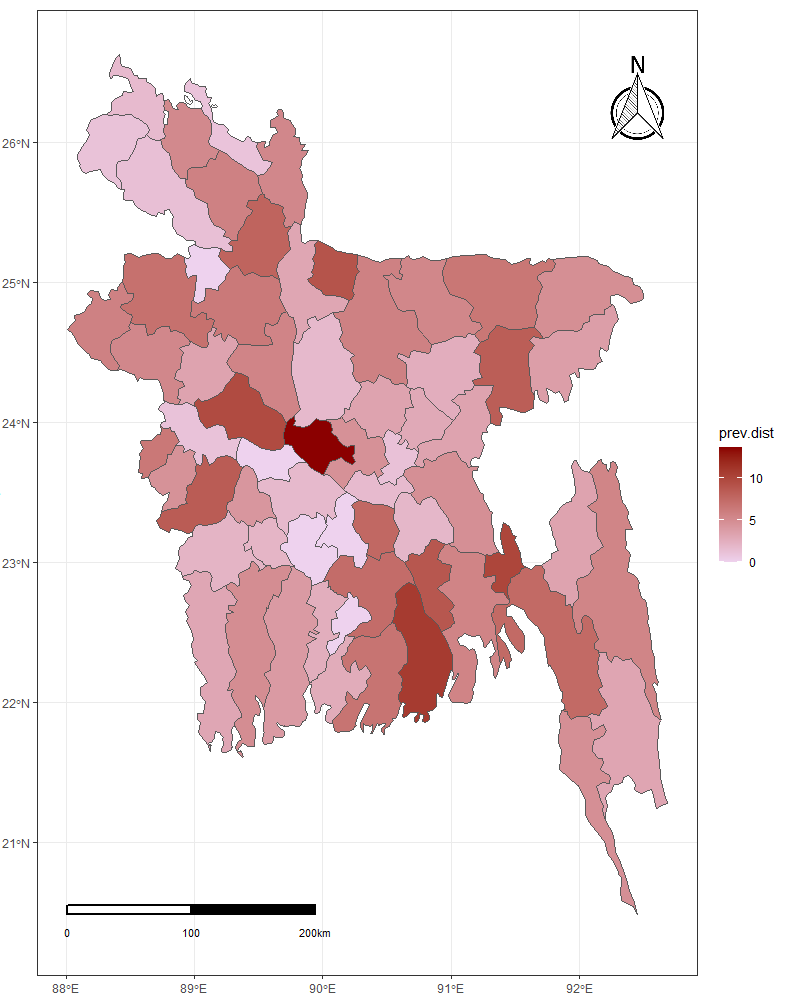


Prevalence

of diarrhea (%)

Fig. S5: The diarrhea prevalence of children under five in 2017-2018

(made by author, Source: BDHS and BARC)


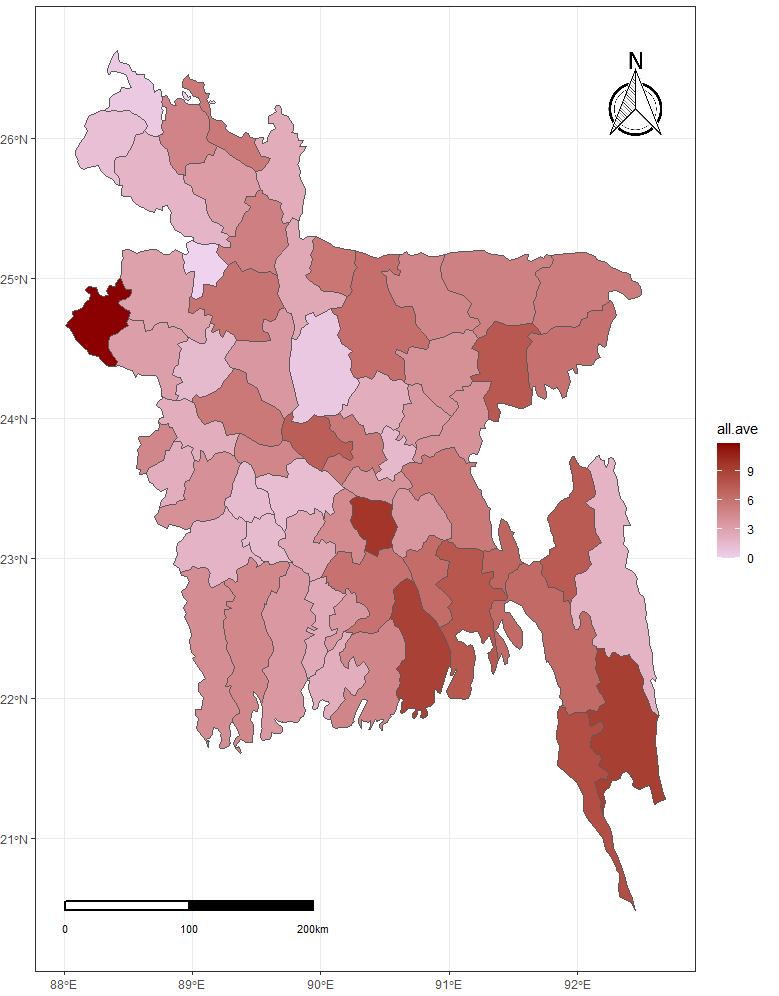


Prevalence

of diarrhea (%)

Fig. S6: The diarrhea prevalence of children under five, sum of DHS2011, 2014 and 2017-2018 (made by author, Source: BDHS and BARC)
